# Chlamydia trachomatis and Neisseria gonorrhoeae bacterial loads in men who have sex with men on pre-exposure prophylaxis: a cross-sectional study

**DOI:** 10.1136/sextrans-2025-056579

**Published:** 2025-08-17

**Authors:** Enrique Rayo, Giulia Malingamba, Hanna Marti, Delia Onorini, Cory Ann Leonard, Nicola Low, Benjamin Hampel, Nicole Borel

**Affiliations:** 1 Chlamydia Group, University of Zurich Institute of Veterinary Pathology, Zurich, Switzerland; 2 Henry M Jackson Foundation for the Advancement of Military Medicine Inc, Bethesda, Maryland, USA; 3 Institute of Social and Preventive Medicine, University of Bern, Bern, Switzerland; 4 Department of Public and Global Health, University of Zurich Institute of Epidemiology Biostatistics and Prevention, Zürich, Switzerland

**Keywords:** Chlamydia Infections, Gonorrhea, Pre-Exposure Prophylaxis, Nucleic Acid Amplification Techniques

## Abstract

**Abstract:**

**Objective:**

*Chlamydia trachomatis* (CT) and *Neisseria gonorrhoeae* (NG) are the most commonly reported sexually transmitted infections globally. Anorectal CT/NG detection among men who have sex with men (MSM) and coinfections is common. Epidemiological studies suggest that CT/NG coinfections might result in greater bacterial load and transmissibility than single infection. The purpose of this study was to compare bacterial load and symptoms between CT/NG single and coinfections in MSM.

**Methods:**

MSM positive for CT or NG on a triple swab (throat, urethra and rectal locations combined) were enrolled. Before treatment, they self-collected anorectal swabs. Bacterial loads for CT/NG were calculated using real-time PCR and compared between single or coinfected individuals, with or without rectal symptoms.

**Results:**

We enrolled 382 MSM from December 2021 to December 2024. Among all samples: total CT (n=114), total NG (n=125), CT/NG coinfection 29/382 (7.6%). The bacterial loads in single and coinfected samples were comparable. The mean difference between CT alone and CT/NG was 0.40 target copies/mL (95% CI (−0.09 to 0.89), p value=0.107). The mean difference for NG alone and CT/NG was 0.24 copies/mL (95% CI (−0.49 to 0.99), p value=0.498). Among 382 MSM, 15.4% (n=59/382) experienced anorectal symptoms. There was no statistical difference in bacterial burdens between symptomatic and asymptomatic (CT difference of the means 0.52 copies/mL, 95% CI (−0.51 to 1.55); p value=0.313) (NG difference of the means 0.63, CI (0.01 to 1.28); p value=0.05).

**Conclusions:**

In contrast to prior research, we found similar bacterial burdens in anorectal MSM samples with single CT/NG versus coinfection. Further research is needed to understand the clinical implications of CT/NG coinfections. Future studies should investigate factors influencing anorectal CT/NG bacterial burden, transmissibility and susceptibility, including the function of pre-exposure prophylaxis and the rectal microbiota.

**Key Messages**

- Among MSM on PrEP, only 15.4% reported anorectal symptoms, and there were no significant differences in bacterial loads between symptomatic and asymptomatic individuals, indicating that symptom presence may not correlate with infection severity in this population.
- This study highlights the importance of considering sexual behaviour, prophylactic drug use, and detailed participant histories in STI research to develop more effective interventions and reduce the burden of CT and NG co-infections, particularly among MSM populations.
- This study found no significant differences in the bacterial load of Chlamydia trachomatis (CT) or Neisseria gonorrhoeae (NG) between single and co-infected MSM using PrEP, challenging previous research that suggested co-infections could exacerbate symptoms and increase bacterial load.

## Introduction

*Chlamydia trachomatis* (CT) and *Neisseria gonorrhoeae* (NG) are the most reported bacterial sexually transmitted infections (STIs) globally.^1^ Coinfections of both pathogens are common, with up to 40% of individuals with NG also having CT. Thus, a biological relationship between CT/NG has been hypothesised, as an increase in CT transmissibility or susceptibility would be required to duplicate reported levels of coinfection. Higher gonococcal loads have been found to increase transmission probability in urogenital infections,^2^ so higher loads in CT/NG coinfections than single infections might result in more symptomatic infections and increase transmissibility. In a retrospective study from the Netherlands, NG bacterial loads were higher in CT/NG coinfection than in NG infection alone in anorectal swabs from men and in vaginal samples from women.^3^ Bacterial loads did not differ between symptomatic and asymptomatic individuals. The study, however, did not report CT loads in single infections or coinfections.

Anorectal CT and NG alone and coinfections are common among men who have sex with men (MSM).^4 5^ Prevalence is even higher among MSM who take pre-exposure prophylaxis (PrEP) to prevent HIV infection,^6^ making them an important subpopulation among whom to investigate the epidemiology and clinical impact of CT/NG coinfections. Here, we aimed to investigate CT and NG anorectal infection load among MSM using PrEP, to compare bacterial loads both in single and coinfections and in symptomatic and asymptomatic infections.

## Methods

Participants in this research were part of the SwissPrEPared Study, at the Checkpoint Clinic Zurich, Switzerland, which provides PrEP and sexual healthcare for people at high risk of acquiring HIV infection (swissprepared.ch). SwissPrEPared participants attend follow-up visits at the clinic every 3 months, which include STI testing. Self-collected swabs from the urethra, anorectal canal and throat are placed in a single container for PCR detection of CT and NG. Those positive for CT and/or NG who returned to the clinic were invited to enrol in our study before antimicrobial treatment. Participants provided a self-collected anorectal swab (Eswab, COPAN, Italy), stored at 4°C and provided information about anorectal symptoms. Staff from our laboratory collected all swabs 24–72 hours postsampling, transported them to the laboratory (2.5 km away) on ice and stored them at −80°C. DNA was extracted from a 200 µL starting volume and eluted in 60 µL (DNeasy Blood and Tissue Kit protocol, Qiagen; Germany). DNA extracts were tested for CT and NG on the Applied Biosystems 7500 Fast Real-Time PCR System, as described previously.^7 8^ CT was detected by targeting the 23S rRNA gene (CT+if Ct <38) and NG using a validated in-house assay targeting the *porA* pseudogene (NG+if Ct <36). Bacterial loads were calculated from standard curves based on 10-fold serial dilutions of synthetic plasmid containing respective target sequences. Amplification efficiencies for both targets were confirmed to be within the range of 90%–110%. All reactions were run in duplicate. Each run included no-template controls and internal inhibition controls using exogenous DNA to verify assay integrity. Bacterial loads for each sample were expressed as log10 copies/mL. We calculated coinfection proportions (with 95% CI), mean bacterial load and SD for single infections, coinfections and the difference between the means (with 95% CI). We compared the difference between the means for CT or NG infection alone and CT/NG coinfection, and for individuals with and without anorectal symptoms, using Tukey’s HSD (honestly significant difference) test in the R statistical software (R Core Team, 2020). All participants provided written informed consent. Ethical approval was granted by the Zurich cantonal ethics committee (project #2021–01802).

## Results

**Figure 1:**
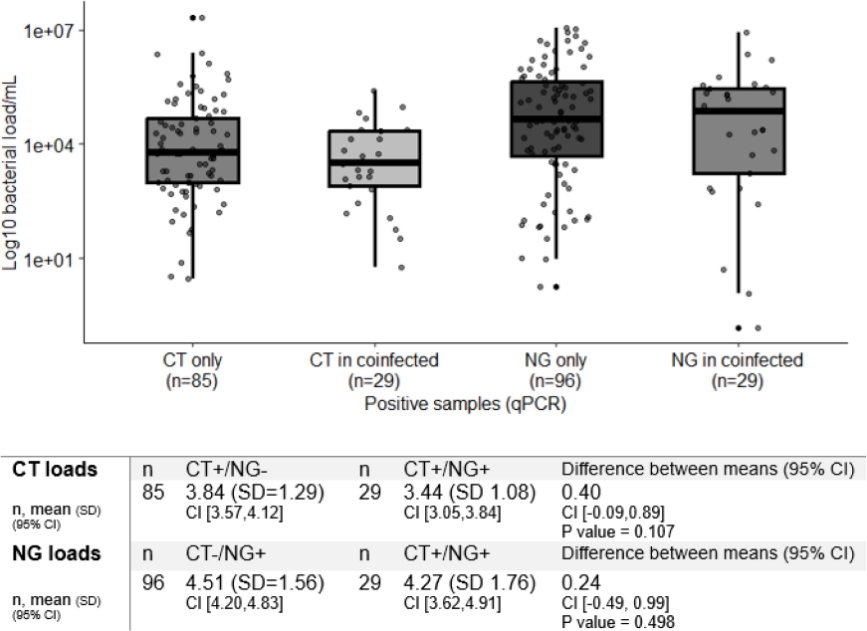
Box plot shows bacterial loads assessed by real-time quantitative PCR assays for *C. trachomatis* and *N. gonorrhoeae* in samples with single and co-infection, and differences (coinfected minus single), tabulated below. The central line within each box represents the median, the top and bottom of each box mark the IQR from the first to the third quartile, capturing the middle 50% of values. Whiskers extend up to 1.5 times the IQR from each quartile, and points beyond the whiskers represent outliers. CT, *C. trachomatis*; NG, *N. gonorrhoeae*.

Between December 2021 and December 2024, we collected 485 anorectal swabs. We excluded non-PrEP users (55 MSM) to ensure population consistency and because the small size of the non-PrEP group limited statistical power for stratified analysis. We also excluded duplicates (47 samples from 42 different MSM, only first sample collected was used), resulting in 382 unique PrEP anorectal swabs. Of these, 22.3% (n=85/382) were positive only for CT and 25.1% (n=96/382) were positive only for NG. In total, 7.6% (n=29/382) were positive for both CT and NG. In single infections, NG loads (4.51, CI (4.20 to 4.83)) were higher than CT loads (3.84, CI (3.57 to 4.12)) (difference between the means 0.67, CI (0.25 to 1.09), p value 0.0019). In coinfections, NG loads (4.27, CI (3.62 to 4.91)) were higher than CT loads (3.44, CI (3.05 to 3.84)) (difference of the means 0.82, CI (0.05 to 1.59), p value 0.037; significant at α=0.05). The CT load in CT/NG coinfection was similar to single infection CT loads (difference of the means 0.40 copies/mL, 95% CI (−0.09 to 0.89), p value 0.107). The NG load in CT/NG coinfection showed no significant difference compared with single infection NG loads (difference of the means 0.24 copies/mL, 95% CI (−0.49 to0.99), p value 0.498) (figure 1).

Among 382 MSM, 15.4% (n=59/382) reported anorectal symptoms. Bacterial loads for CT did not differ between symptomatic and asymptomatic individuals (CT copies in symptomatic 4.09, SD 1.74; CT copies in asymptomatic 3.57, SD 1.29; differences between the means 0.52, 95% CI(−0.51 to 1.55), p value 0.313). NG loads in symptomatic individuals tended to be higher than in asymptomatic individuals, but the difference did not reach statistical significance (NG copies in symptomatic 4.67, SD 1.59, NG copies in asymptomatic 4.04, SD 1.79; difference between the means 0.63, 95% CI (0.01 to 1.28), p value 0.05).

## Discussion

In this cross-sectional study among MSM in the SwissPrEPared study, we did not find statistical evidence of a difference in bacterial loads in CT/NG anorectal coinfections compared with either CT or NG alone or in symptomatic compared with asymptomatic infections.

Our results for NG loads in anorectal swabs in men differ from those reported by van Dessel *et al*, who found higher NG loads in CT/NG coinfected samples (mean 4.70 log_10_ copies/mL, 95% CI 2.21 to 6.39, n=210) than in samples with NG alone (4.09, 1.57 to 6.64, n=825).^3^ In our study, the mean NG load in CT/NG coinfection was slightly lower than that in single infection (figure 1). The reason for the different findings might result from differences between participants in the two studies. Our study included only MSM taking PrEP for HIV prevention, where participants receive frequent healthcare visits. Frequent STI testing is likely to detect newly acquired infections, which may be associated with smaller difference in bacterial load between single and coinfection. In the study by van Dessel *et al,* information about PrEP use and time from infection to sampling were not reported. The van Dessel study also reported that there was no difference in a smaller sample of anorectal swabs from women. Mean load in coinfection (4.42, 2.18 to 6.50, n=35) was, however, higher than in single infection (3.81, 1.82 to 5.95, n=105). For both men and women, the difference between the means is 0.61 log_10_ copies/mL. In both studies, the presence or absence of symptoms was not associated with a difference in bacterial load.

Although higher bacterial loads have been associated with increased transmission in urogenital gonorrhoea,^2^ the clinical relevance of bacterial burden in anorectal infections remains unclear. Among those with NG, the proportion of anorectal CT/NG coinfections is not well described but might be less common than vaginal coinfections. Among anorectal samples with NG detected, the proportions with both CT and NG are similar among men in our study (23%), and both men (20%) and women (25%) in the study by van Dessel *et al.* In vaginal samples from the study by van Dessel *et al,* 40% of samples with NG (n=349) also had CT detected (n=142). This proportion is consistent with other studies of women with NG.^9 10^ While bacterial loads may reflect transmissibility or correlate with the likelihood of symptomatic presentation, current evidence for their clinical utility—particularly in anorectal CT/NG infections—is limited, inconsistent, and lacks standardised interpretive cut-offs. Future studies should investigate CT/NG coinfections in a range of anatomical sites in both men and women and in those with both CT and NG. Factors of relevance include the role of medication including PrEP, viability or non-viability of PCR-detected pathogens, and the potential role of other concomitant bacteria.

The strengths of our study include the structured study design, which allowed consistent collection of samples over time. Sample collection before antibiotic treatment ensured that loads were all from untreated infections. Our study has some limitations. The sample size was relatively small, and we focused exclusively on anorectal infections among MSM on PrEP, as this site allows for more reliable bacterial load quantification and symptom assessment, but limits generalisability. The limited number of coinfections (n=29) reduces the precision of bacterial load estimates. However, we believe it is unlikely that we missed a clinically significant difference, as the differences between the means were minimal (figure 1).

## Conclusion

In this study, *C. trachomatis* and *N. gonorrhoeae* bacterial loads did not differ between coinfections and single infections in MSM, in contrast to earlier studies. These findings challenge the assumption that coinfection is associated with elevated bacterial burden. Further research is needed to clarify the biological interactions between these pathogens in coinfected individuals.
